# DNA Methylation-Mediated Regulation of Plant Adaptation to Abiotic Stress

**DOI:** 10.3390/plants15203166

**Published:** 2026-10-16

**Authors:** Guan Liu, Hanhui Wang, Yifei Tang, Huan Gao, Yang Wang, Yan Sun, Song Yu, Dongye Zhang, Zizhu Jiang

**Affiliations:** 1 College of Advanced Agriculture and Ecological Environment, Heilongjiang University, Harbin 150006, China; 2019057@hlju.edu.cn (G.L.); 18263417429@163.com (Y.T.); 2022112@hlju.edu.cn (H.G.);; 2 State Key Laboratory of Tree Genetics and Breeding, College of Forestry, Northeast Forestry University, Harbin 150040, China; 3 Department of Architecture and Urban Planning, Shandong Urban Construction Vocational College, Jinan 250103, China; 18846938745@163.com

**Keywords:** DNA methylation, abiotic stress, plant adaptation, epigenetic regulation

## Abstract

Climate change is intensifying environmental stresses, including drought, high temperature, and salinity, posing increasing challenges to plant growth and development. In addition to genetic variation, DNA methylation is an important epigenetic mechanism that regulates gene expression without altering the underlying DNA sequence through the addition of methyl groups to the fifth carbon of cytosine. Genome-wide dynamic changes in DNA methylation and demethylation at CG, CHG, and CHH sites contribute to the regulation of stress-responsive transcriptional networks, thereby shaping plant phenotypes and facilitating the establishment of stress memory. In recent years, advances in methylome profiling and molecular validation have shifted research from describing DNA methylation changes to elucidating their regulatory mechanisms. In this review, we summarize the molecular mechanisms underlying the establishment, maintenance, and removal of DNA methylation, with a particular focus on its dynamic changes and regulatory roles in plant responses to cold, heat, drought, and salt stresses. It also compares the characteristics of DNA methylation responses across different types of stress and discusses their potential molecular mechanisms. Overall, DNA methylation represents an important regulatory layer in plant responses to abiotic challenges, and its dynamic changes are closely associated with plant adaptation, providing new insights into the mechanisms underlying stress adaptation.

## 1. Introduction

Plant adaptation to environmental stresses is a gradual process that involves a transition from short-term phenotypic plasticity to long-term adaptation. In the short term, plants rapidly respond to and mitigate environmental stresses through morphological, physiological, and biochemical adjustments. As stress persists, some beneficial changes may be maintained and eventually contribute to the formation of stable adaptive traits [1,2,3]. In this process, epigenetic modifications serve as important regulatory mechanisms linking environmental signals to genomic responses. Epigenetic mechanisms, including DNA methylation and histone modifications, can respond to environmental changes and dynamically regulate gene expression without altering the underlying DNA sequence, thereby contributing to plant stress responses and adaptation [4,5]. The emerging concept of “epigenetic memory” further suggests that some stress-induced epigenetic changes can persist after the removal of stress and may be transmitted to subsequent generations, thereby conferring more sustained adaptive capacity to plants [6,7,8].

DNA methylation is one of the earliest discovered and most extensively studied epigenetic modifications. It refers to the addition of a methyl group to the fifth carbon atom of cytosine and represents a highly conserved epigenetic mark in eukaryotes [9,10]. DNA methylation plays critical roles in maintaining genome stability, mediating transposable element (TE) silencing, and regulating gene expression [11,12]. Unlike mammals, in which DNA methylation predominantly occurs in the CG sequence context, plants exhibit a more complex sequence-dependent methylation pattern, mainly comprising three contexts: CG, CHG, and CHH (H = A, T, or C) [10]. In addition, DNA methylation has distinct functions depending on its genomic location. High methylation levels in promoter regions are generally associated with transcriptional repression, whereas CG methylation within gene bodies has been shown to be associated with transcriptional elongation and alternative mRNA splicing [13,14,15]. In contrast, high methylation levels in transposable elements and repetitive sequences contribute to the maintenance of condensed chromatin states and suppression of transposable element activity, thereby helping to preserve genome stability [12].

Numerous studies have demonstrated that DNA methylation is involved in plant responses to various abiotic stresses, with its establishment, maintenance, and removal being precisely regulated by multiple DNA methyltransferases, demethylases, and associated regulatory factors [12,16]. Under stress conditions, the plant methylome undergoes dynamic reprogramming, with specific promoter, gene body, and transposable element regions exhibiting hypermethylation or hypomethylation. These changes can regulate stress-responsive gene expression networks, transposable element activity, and chromatin states, thereby influencing plant stress phenotypes and stress memory [17,18]. Although the importance of DNA methylation in plant stress responses has been widely recognized, the patterns of methylation changes under different stress conditions remain unclear, and the molecular mechanisms by which DNA methylation regulates the expression of stress-responsive genes require further investigation. In this review, we primarily focus on recent studies, with an emphasis on representative studies that elucidate the specific molecular mechanisms underlying the involvement of DNA methylation in plant stress responses, while also retaining earlier studies that provide foundational or mechanistic insights. Priority is given to studies that provide evidence linking changes in DNA methylation with target gene expression and functional validation, thereby highlighting the recent shift in this field from descriptive observations toward mechanistic understanding. We introduce the molecular mechanisms underlying the establishment, maintenance, and removal of DNA methylation, with particular emphasis on its dynamic patterns and regulatory roles in plant responses to heat, cold, drought, and salt stresses. The characteristics of DNA methylation responses under different stresses are further summarized, together with their potential molecular mechanisms. Thus, DNA methylation represents an important regulatory layer in plant responses to abiotic stresses, and its dynamic patterns are closely associated with plant adaptation and stress tolerance, providing new insights into the molecular mechanisms underlying stress adaptation.

## 2. Regulation of Plant DNA Methylation

### 2.1. Key Regulatory Factors of Plant DNA Methylation

**Figure 1:**
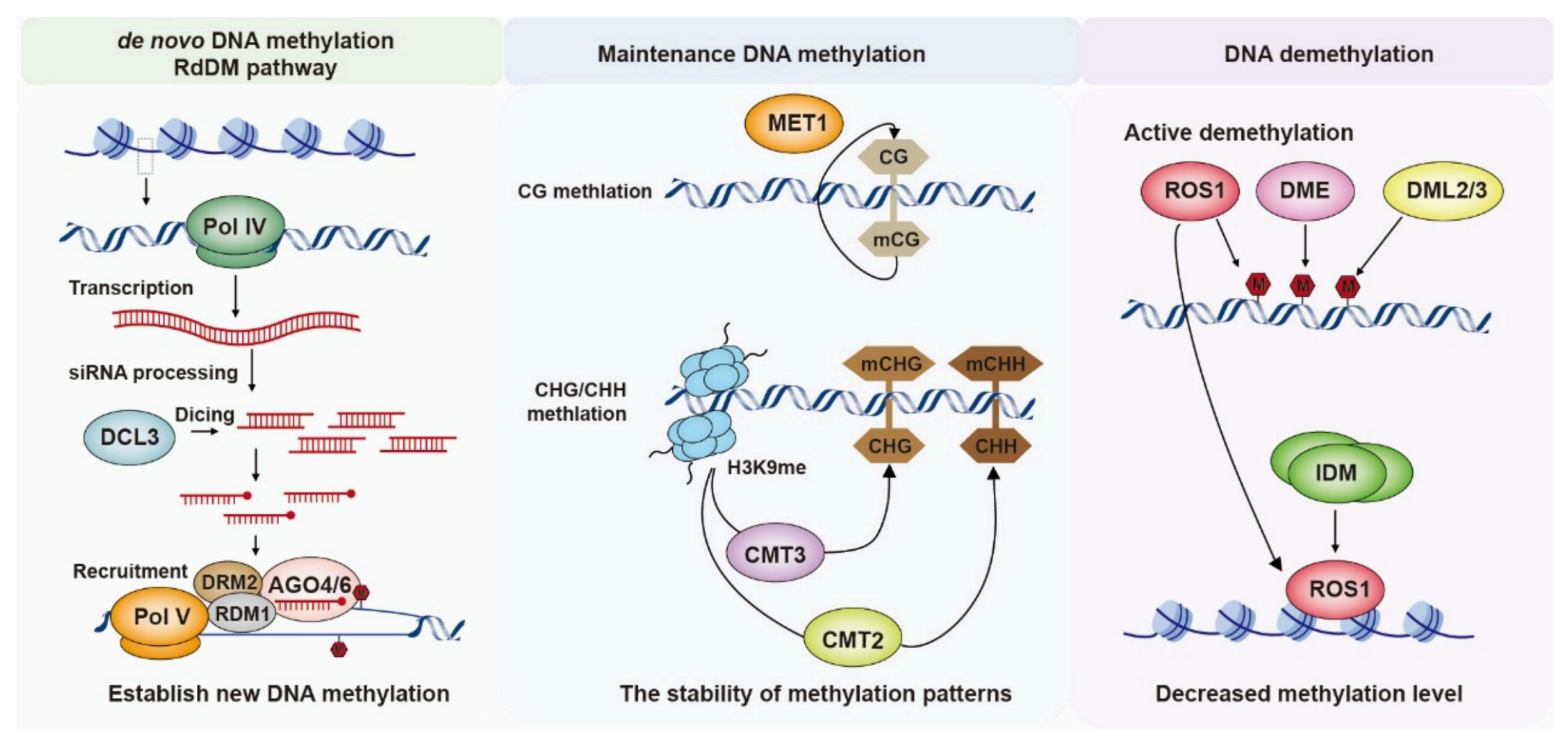
Molecular mechanisms of DNA methylation establishment, maintenance, and demethylation in plants. The de novo DNA methylation pathway is mediated by RNA-directed DNA methylation (RdDM), in which RNA polymerase IV (Pol IV) and RNA polymerase V (Pol V), together with DCL3, AGO4/6, RDM1, and DRM2, establish new DNA methylation. Maintenance of DNA methylation involves MET1-mediated CG methylation and CMT3/CMT2-mediated CHG/CHH methylation, which is associated with H3K9me. DNA demethylation occurs through active demethylation mediated by ROS1, DME, and DML2/3, with IDM facilitating ROS1 recruitment and subsequent removal of methylated cytosines.

DNA methylation primarily occurs at the 5′ position of cytosine, with 5-methylcytosine (5mC) being the most common form. Across plant genomes, methylated cytosines generally account for approximately 5–50% of all cytosines [19,20]. Plant DNA methylation is not static but is dynamically regulated through processes of establishment, maintenance, and removal, thereby maintaining relatively stable methylation levels and enabling plants to adapt to different developmental stages and environmental conditions [21,22,23]. This dynamic balance is primarily regulated by the coordinated actions of DNA methyltransferases and DNA demethylases (Figure 1) [9,17].

Major plant DNA methyltransferases include DNA methyltransferase 1 (MET1), domain-rearranged methyltransferases (DRMs), and chromomethylases (CMTs) [24,25]. MET1 is mainly responsible for the maintenance of CG methylation [26], whereas DRMs primarily mediate de novo DNA methylation through the RNA-directed DNA methylation (RdDM) pathway, establishing new DNA methylation in different sequence contexts and playing a particularly important role in the establishment of CHH methylation [27]. CMTs are mainly involved in the establishment and maintenance of non-CG methylation, with CMT3 primarily responsible for the maintenance of CHG methylation and CMT2 mainly involved in the maintenance of CHH methylation [28]. In contrast to the establishment and maintenance of DNA methylation, active DNA demethylation in plants is primarily mediated by DNA demethylases, including REPRESSOR OF SILENCING 1 (ROS1), DEMETER (DME), DEMETER-like 2 (DML2), and DML3, which collectively regulate DNA methylation levels in plants [29].

### 2.2. Establishment, Maintenance, and Removal of DNA Methylation

The formation and stability of plant DNA methylation are primarily governed by three interconnected processes: establishment, maintenance, and removal (Figure 1) [22,20]. First, de novo DNA methylation is mainly mediated by the RdDM pathway. In this process, RNA polymerase IV (Pol IV) transcribes RNA precursors, which are processed into 24-nt small interfering RNAs (siRNAs). These siRNAs are subsequently loaded onto ARGONAUTE (AGO) proteins and participate in target recognition, ultimately recruiting the DRM2 methyltransferase to establish new DNA methylation at target sites [30].

Once established, DNA methylation must be maintained through distinct mechanisms to ensure the stability of methylation patterns [22]. The maintenance of CG methylation primarily depends on MET1, which recognizes hemimethylated DNA generated during DNA replication and restores methylation on the newly synthesized strand, thereby maintaining the CG methylation pattern. In contrast, the maintenance of non-CG methylation mainly depends on CMT2 and CMT3, which interact with histone modifications such as H3K9me to form mutually reinforcing feedback loops, thereby maintaining CHG and CHH methylation states [31,32].

Accordingly, plants can also reduce DNA methylation levels in specific genomic regions through passive and active demethylation. Passive DNA demethylation occurs during DNA replication, when impaired or inhibited maintenance methylation mechanisms, such as those mediated by MET1, prevent 5mC from being effectively maintained on the newly synthesized DNA strand, leading to its gradual dilution through successive rounds of replication [33]. In contrast, active DNA demethylation directly removes 5mC from DNA through enzymatic processes and is mainly mediated by DNA demethylases, including ROS1, DME, DML2, and DML3, which specifically recognize and remove 5mC [34,35,36]. Among these enzymes, ROS1 is an important mediator of active DNA demethylation in plants. Its targeting is coordinated by IDM protein complexes and associated chromatin modifications, enabling DNA demethylation to occur precisely at specific genomic regions [35].

## 3. DNA Methylation-Mediated Regulation of Plant Responses to Abiotic Stress

### 3.1. Temperature Stress

DNA methylation changes in response to high temperature are not simply manifested as a global increase or decrease in genome-wide methylation levels. Recent studies have shown that high temperature does not necessarily induce extensive genome-wide DNA methylation changes, but rather causes dynamic remodeling at specific loci and genomic regions (Table 1). For example, increased temperatures (28 °C) induce genome-wide hypermethylation in broccoli (*Brassica oleracea*) compared with the control temperature of 16 °C, whereas strawberry (*Fragaria vesca*) exhibits a slight reduction in DNA methylation, and rapeseed (*Brassica napus*) exhibits an overall reduction in DNA methylation under high-temperature stress [37,38,39]. In *Arabidopsis thaliana*, overall DNA methylation changes during heat stress are relatively limited, whereas site-specific active demethylation at CG, CHG, and CHH sites occurs predominantly during the recovery phase after heat stress removal. These changes are mainly distributed within gene-coding regions and are enriched in stress-related genes involved in heat shock response, oxidative stress, and drought response [40]. Similarly, heat stress in diploid woodland strawberry (*Fragaria vesca*) causes only minor changes in genome-wide DNA methylation, with mCG levels decreasing by approximately 3.2%, but induces numerous DMRs, predominantly characterized by CHH hypomethylation and enriched in centromeric/pericentromeric regions and promoters. Notably, pronounced CHH demethylation occurs in MITE transposable elements and in the promoters of heat-responsive transcription factors, including AP2/EREBP, HSF, and WRKY [38]. In *A. thaliana*, heat stress also activates the LTR retrotransposon ONSEN (ATCOPIA78), accompanied by reduced CHH methylation and enhanced transcription. Meanwhile, heat-induced 24-nt siRNAs and RdDM-associated factors, including the DDR complex and SUVH2, are associated with the restriction of ONSEN transposition, suggesting a dual-layer “transcriptional activation–transposition suppression” defense mechanism [41,42]. In addition, HD2B/HD2C-mediated histone deacetylation acts cooperatively with the AGO4-RdDM pathway to maintain heterochromatin stability. Disruption of this regulatory network by heat stress reduces siRNA abundance and CHH methylation, and is accompanied by heterochromatin relaxation and reduced heat tolerance [43]. Under long-term cyclical heat stress, MET1-, CMT3-, and DRM2-mediated DNA methylation-dependent silencing also cooperates with MOM1-dependent non-methylation-mediated silencing. These pathways may contribute to restricting aberrant transposon activation and maintain genome stability [44].

Methylation remodeling under different genetic backgrounds further determines plant heat tolerance and plays an important role in reproductive development. In cotton (*Gossypium hirsutum*), heat stress suppresses the expression of core RdDM genes, including RDR2, DCL3, AGO4, and DRM2, in heat-sensitive genotypes, resulting in an abnormal reduction in CHH methylation in anthers, which is accompanied by disruptions in sugar metabolism and ROS production, as well as microspore abortion and anther indehiscence. In contrast, heat-tolerant genotypes maintain relatively high levels of CHH methylation, which is associated with greater heat tolerance [45]. Studies in different maize (*Zea mays*) inbred lines have shown that transposon responses to heat stress are highly genotype-dependent, with their transcriptional potential closely associated with pre-existing unmethylated regions (UMRs). Transposons retaining UMRs are more readily induced by heat stress, whereas highly methylated transposons remain transcriptionally silenced [46]. Recent studies in rice (*Oryza sativa* L.) further revealed that tetraploid rice exhibits more pronounced genome-wide and stress-responsive locus-specific DNA demethylation than diploid rice, accompanied by enhanced expression of genes involved in hormone signaling, ROS scavenging, and heat shock responses. These methylation and transcriptional changes were associated with reduced ROS accumulation, improved water retention, and stress recovery. [47]. Heat tolerance does not simply depend on the overall level of DNA methylation, but rather may be associated with the precise remodeling of specific methylation sites and their coordinated association with stress-responsive genes, ROS homeostasis, and reproductive development. In particular, RdDM-mediated CHH methylation reprogramming and its spatiotemporal specificity may represent an important epigenetic hub linking heat stress signals to plant heat-tolerance phenotypes.

Additionally, DNA methylation is one of the most extensively studied epigenetic modifications in plant responses to cold stress, and its dynamic changes are closely associated with the transcription of cold-responsive genes, plant growth and development, and cold tolerance [18,48,49]. DNA methylation responses vary among plant species. Global DNA methylation levels decrease following cold treatment in sugar beet (*Beta vulgaris*) [50] and poplar (*Populus* L.) [51], whereas no significant overall changes are observed in apple (*Malus × domestica* Borkh.) [52] between field sites at different altitudes, where temperature conditions differed but were not experimentally controlled. In rubber tree (*Hevea brasiliensis*) [53], *Brassica rapa* [54], tomato (*Solanum lycopersicum)* [55], and sweet orange (*Citrus sinensis* L.) [56], promoter demethylation of cold-responsive genes is associated with increased transcription of these genes. Genotype-dependent differences in DNA methylation are also evident. The cold-tolerant rice cultivar P427 exhibits more pronounced CHH demethylation under low-temperature conditions, with reduced methylation in the *Os03g0610900* promoter accompanied by increased expression of *Os03g0610900*, an OST1 homolog encoding a SnRK2 family protein kinase involved in ABA signaling and stress responses. These changes are associated with the activation of the ICE-CBF-COR cold-response pathway and enhanced antioxidant and ABA responses [57]. In sugarcane, cold stress increases CpG island methylation in the *ScMYB7* promoter, which is associated with reduced expression of this negative regulator of cold tolerance, along with changes in the CBF/DREB pathway and downstream COR gene expression [58]. In tea plants, transposon-mediated methylation changes are associated with changes in the local methylation status and expression of duplicated genes, which may contribute to gene divergence and adaptation to cold stress [59].

More importantly, some cold-induced DNA methylation changes can persist after stress removal and may contribute to stress memory and even heritable epigenetic states, although their persistence across generations is variable. In cucumber, cold treatment increases genome-wide DNA methylation, which largely returns to basal levels after rewarming, while methylation at certain loci persists and is accompanied by incomplete recovery of radicle growth, suggesting an association between residual methylation and cold stress memory [60]. In rice, repeated exposure to low temperature during meiosis is associated with downregulation of *MET1b*, CG/CHG demethylation at the *ACT1* promoter of the cold-sensitive rice cultivar KD8, and sustained *ACT1* expression under cold conditions. These changes are associated with improved panicle fertility and seed-setting rate, and this process ultimately generates a stably inherited epiallele associated with cold tolerance [61]. However, not all stress-induced methylation changes are stably inherited across generations, as some may be reset during germline development, and distinguishing true epimutations from changes associated with underlying genetic variation remains challenging.

**Table 1:** DNA methylation and epigenetic regulation in plants under temperature stress.

Stress Type	Species	Tissue	Treatment	Method	Evidence Type	Methylation Change	Genes/Pathways Involved	References
Heat	*Brassica napus*	Cultured microspores	6 h in-vitro incubation at 32 °C/18 °C	Whole-genome bisulfite sequencing (WGBS), pyrosequencing, qRT-PCR	Descriptive methylome	Global DNA methylation decreases	Stress-responsive genes	[37]
Heat	*Fragaria vesca*	Above-ground tissues of one-month-old seedlings	37 °C two weeks	WGBS, RNA-seq	Descriptive methylome	A slight reduction in DNA methylation, but numerous DMRs (predominantly CHH hypomethylation)	*AP2/EREBP*, *HSF*, *WRKY*; MITE transposons	[38]
Heat	*Brassica oleracea* cv. Green Harmony F1	Shoot apex tissues	16 °C/22 °C/28 °C, 0.2 mM 5-azaC at 28 °C	WGBS, RNA-seq, 5-azaC chemical inhibitor assay, qRT-PCR	Pharmacological perturbation	Global hypermethylation	Ribosome biogenesis, RNA metabolism, and transcriptional regulatory pathways	[39]
Heat	*Arabidopsis thaliana*	Seedling leaves	42 °C heat stress for 24 h	WGBS	Descriptive methylome	Site-specific active demethylation at recovery phase	Heat shock, oxidative stress, drought-responsive genes	[40]
Heat	*Arabidopsis thaliana*	7-day-old seedlings	37 °C heat stress for 24 h or 48 h	WGBS, Chop-PCR, Bisulfite Sanger sequencing, mutant genetic analysis	Epigenetic validation	Reduced CHH methylation at LTR retrotransposon ONSEN	*DDR*, *SUVH2*; *HD2B/HD2C*, *AGO4*; ONSEN transposon	[41,42]
Heat	*Arabidopsis thaliana*	18-day-old seedling leaves	44 °C for 3 h then recovery, 37 °C for 24 h for WGBS	WGBS, RNA-seq, ChIP-seq, McrBC-qPCR, mutant genetic analysis	Epigenetic validation	Significant decrease in CHH methylation	HD2B, HD2C, AGO4, RdDM pathway	[43]
Heat	*Arabidopsis thaliana*	Whole seedlings	37 °C day/21 °C night, long-term cyclical heat stress	*met1-1*, *drm2-2/cmt3-11*, *mom1-2/mom1-3* mutants, RNA-seq	Expression correlation	MET1-, CMT3- and DRM2-mediated methylation-dependent silencing	*MET1*, *CMT3*, *DRM2*, *MOM1*	[44]
Heat	*Gossypium hirsutum*	Anthers	day 39–41 °C/night 29–31 °C, 150 μM Zebularine for methylation inhibition	WGBS, RNA-seq, small-RNA-seq, Zebularine treatment	Pharmacological perturbation	Abnormal reduction in CHH methylation in anthers in heat-sensitive genotype, maintains relatively high CHH methylation levels in heat-tolerant genotype	*RDR2*, *DCL3*, *AGO4*, *DRM2*	[45]
Heat	*Zea mays*; different inbred lines	13-day-old seedling third-leaf tissue	heat stress 42 °C for 4 h	WGBS, RNA-seq	Expression correlation	Genotype-dependent and UMR-associated transposon responses	Transposons (UMRs)	[46]
Heat	*Oryza sativa*	Three-leaf-stage seedling leaf tissues	heat-treated at 40 °C for 7 d, followed by 7-d recovery	WGBS (MethylC-seq), RNA-seq	Expression correlation	Tetraploid shows more pronounced genome-wide and stress-responsive locus-specific DNA demethylation	Hormone signaling, ROS scavenging, heat shock-responsive genes	[47]
Cold	*Beta vulgaris*	Leaf tissues	7 d at 12 °C, 13 d at 4 °C, 24 h at 0 °C	WGBS, RNA-seq	Expression correlation	Global DNA methylation decreases	RdDM pathway	[50]
Cold	Poplar	Apical bud/shoot apical meristem	8 h-light, 19 °C, then 4 °C 8 weeks	WGBS, RNA-seq, HPLC, RNAi transgenic lines	Genetic validation	Global DNA methylation decreases	Protein homeostasis, chloroplast metabolism, mitochondrial transport	[51]
Different altitudes	*Malus domestica*	Floral spur bud	high-chill site (altitude 2350 m); low-chill site (altitude 1250 m)	Bisulfite sequencing, RNA-seq, MSAP	Expression correlation	No significant overall changes	—	[52]
Cold	*Hevea brasiliensis*	Leaf tissues	short-term cold: 19 °C or 4 °C, 24 h; long-term cold: 19 °C for 1 month	Bisulfite sequencing, qRT-PCR	Expression correlation	Promoter demethylation of cold-responsive genes	HbICE1, HbCBF1-3, HbMET, HbCMT, HbDRM, etc.	[53]
Cold	*Brassica rapa*	The third fully expanded leaf	4 °C, 16 h light/8 h dark, 2 weeks	Bisulfite sequencing, MeDIP-seq, RNA-seq/qRT-PCR, 5-Aza treatment	Pharmacological perturbation	Promoter demethylation of cold-responsive genes	BramMDH1, BraKAT2, BraSHM4, Bra4CL2	[54]
Cold	*Solanum lycopersicum*	Pericarp tissue	7 d at 5 °C followed by 1 d recovery at 20 °C	WGBS, RNA-seq, GC-MS	Expression correlation	Promoter demethylation of cold-responsive genes	Fruit ripening transcription factor, and cold signaling pathway	[55]
Cold	*Citrus sinensis*	Flesh segments of post-harvest fruit	4 °C, 25 d	McrBC-qPCR, qRT-PCR	Expression correlation	Promoter demethylation of cold-responsive genes	Flavonoid pathway, and demethylase	[56]
Cold	Oryza sativa	Seedling leaf tissues	2–3 °C, 3 d	MeDIP-seq, RNA-seq	Expression correlation	More pronounced CHH demethylation in cold-tolerant cultivar P427	Os03g0610900; *ICE-CBF-COR*; ABA signaling genes	[57]
Cold	*Saccharum officinarum*	Leaf/root tissues	4 °C, 24 h	Bisulfite sequencing, qRT-PCR, Overexpressed transgenic lines of *A. thaliana*	Expression correlation	Cold-induced ScMYB7 promoter CpG hypermethylation	*ScMYB7*; *CBF/DREB*; *COR*	[58]
Cold	*Camellia sinensis*	Young seedling leaf tissues	4 °C, 7 d	WGBS, RNA-seq, qRT-PCR, MethyKit	Expression correlation	Transposon-mediated methylation changes	Cold signaling pathway, RdDM pathway, transposons	[59]
Cold	*Cucumis sativus*	Seedling radicle	2.5 °C, 3 d; 8 μM 5-azacytidine treatment	MSAP, 5-Aza treatment	Pharmacological perturbation	Cold-induced hypermethylation, mostly reversible but with residual loci	Cell elongation, cell cycle-related pathways	[60]
Cold	*Oryza sativa*	Meiotic-stage young panicle	15 °C, 7 d	WGBS, RNA-seq, qRT-PCR, Chop-PCR, ChIP-qPCR, Bisulfite Sanger sequencing, CRISPR-dCas9 epigenetic editing	Genetic/epigenetic validation	Downregulation of MET1b induces CG/CHG demethylation in the ACT1 promoter	*MET1b*; *ACT1*	[61]

Note: Descriptive methylome refers to genome-wide or methylome profiling without direct functional validation; expression correlation refers to associations between methylation changes and gene expression without direct perturbation; pharmacological perturbation refers to studies using DNA methylation inhibitors such as 5-azacytidine or zebularine; and genetic/epigenetic validation refers to evidence obtained through genetic manipulation or targeted epigenetic editing.

### 3.2. Drought Stress

Recent studies have shown that CHH methylation is often among the most responsive methylation contexts to drought stress and genotypic differences, although its relatively low baseline level may affect the detection of methylation changes. These differentially methylated regions (DMRs) are widely distributed across promoters, gene bodies, and transposable elements and may contribute to drought adaptation through their association with the expression of stress-responsive genes (Table 2). For example, drought stress in mulberry (*Morus alba*) causes an overall increase in genome-wide DNA methylation, primarily attributable to increased mCG levels, with differentially methylated genes mainly involved in autophagy, MAPK, AMPK, carbon metabolism, and amino acid biosynthesis [62]. In linseed (*Linum usitatissimum* L.), drought and repeated drought stress induce dynamic changes in CG, CHG, and CHH methylation, with the drought-tolerant cultivar Z141 exhibiting more hypermethylated regions. The associated genes are mainly involved in carbohydrate and lipid metabolism, ABA signaling, osmotic regulation, and antioxidant responses [63]. In contrast, drought-stressed white poplar (*Populus tomentosa*) shows an overall decrease in 5mC levels, with the most pronounced changes occurring in the CHH context, despite its relatively low basal methylation level. The identified DMRs are predominantly concentrated in transposable element regions and are associated with genes such as *PtoGATA9* and *LECRK-VIII.2*, which are involved in the regulation of stomatal morphology and chlorophyll content [64].

Moreover, differences in DNA methylation patterns are associated with the differentiation of drought-tolerant genotypes. In rice (*Oryza sativa* L.), the drought-tolerant cultivar Nagina 22 exhibits extensive CHH demethylation under dehydration stress, accompanied by the enhanced expression of drought-responsive genes, including bZIP, NAC, AP2, ZEP, and cysteine protease inhibitors, whereas the drought-sensitive cultivar IR64 shows relatively limited methylation changes [65]. These differences in methylation and gene expression patterns are associated with the contrasting drought responses of the two cultivars. Similarly, drought induces an increase in CHH methylation and an enrichment of hypermethylated DMRs in the drought-tolerant wild type mungbean 61 (*Vigna radiata* L.), whereas the drought-sensitive cultivator 70 predominantly exhibits decreased CHH methylation and hypomethylated DMRs, with differentially methylated genes mainly involved in carbohydrate metabolism, signal transduction, and RNA metabolism [66]. In maize (*Zea mays* L.), drought stress generally increases genome-wide DNA methylation, but drought-sensitive genotypes produce substantially more hypermethylated DMRs than drought-tolerant genotypes, with promoter methylation changes associated with genes involved in osmotic stress, oxidative stress, water and salt responses, and RNA silencing [67]. In wheat (*Triticum aestivum* L.), both the drought-tolerant cultivar Bolani and the drought-sensitive cultivar Sistan exhibit genome-wide demethylation. However, Bolani shows demethylation at stress-responsive genes such as PEPC, BGlu, GST, and LSD, which is associated with increased expression of these genes and changes in ABA signaling, antioxidant defense, and photosynthetic protection. In contrast, Sistan shows persistent demethylation, while the two genotypes differ in their ABA-mediated defense responses [68]. Taken together, these observations suggest that drought-tolerant genotypes is not simply associated with either “higher” or “lower” DNA methylation, but rather with distinct and dynamic methylation patterns that coincide with changes in multiple drought-response pathways to establish epigenetic regulatory networks distinct from those of drought-sensitive genotypes.

Beyond genome-wide changes in DNA methylation, recent studies have further revealed locus-specific associations between DNA methylation and the expression of key drought-responsive genes. *ZmNAC111* in maize and *MdRFNR1* in apple (*Malus domestica*) are representative examples of transposon-mediated methylation regulation associated with natural variation in drought tolerance [69,70]. An 82-bp MITE insertion in the promoter of *ZmNAC111* is linked to the generation of 24-nt siRNAs and RdDM pathway activity, alongside increased CG and CHH methylation and enrichment of H3K9me2 at the insertion site. These epigenetic changes coincide with reduced *ZmNAC111* expression and altered ABA-associated drought responses [69]. In contrast, a 430-bp MITE insertion in the promoter of *MdRFNR1-1* in apple is linked to CG, CHG, and CHH hypermethylation and 24-nt siRNA-mediated RdDM activity under drought stress, alongside increased MdRFNR1 expression, higher POD and CAT activities, and greater ROS scavenging [70]. In addition, drought could promote *ZmEXPB2* expression in maize through CG/CHG demethylation and CHH hypermethylation in the middle region of its promoter, which may facilitate primary root elongation and root architectural remodeling [71]. The *HvDME* promoter in barley also exhibits region-specific methylation patterns, with drought-induced increases in CG and CHG methylation and a decrease in CHH methylation occurring specifically in the distal regulatory region, where these cis-acting methylation changes may regulate *HvDME* expression and contribute to a feedback regulatory mechanis [72].

DNA methylation dynamics also contribute to drought stress memory and adaptation to recurrent stress. Drought priming can alter the balance between DNA methyltransferases and demethylases, which may be accompanied by persistent epigenetic changes. For example, drought priming in wheat is accompanied by increased *TaDME* expression and decreased expression of *TaMET2a/2b/3*. CG/CHG demethylation also occurs in specific regions of the *TaP5CS* and *TaBADH* promoters, alongside enhanced osmolyte biosynthesis, which may contribute to improved tolerance to recurrent drought. Moreover, the optimal priming intensity varies among cultivars [73]. In perennial fruit trees, prolonged mild drought is also accompanied by reduced expression of demethylases such as *CsDME* and *CsDML* in Citrus plants. In *Satsuma mandarin*, this response coincides with genome-wide hypermethylation dominated by CHH methylation, as well as silencing of the flowering-repressor genes *FLC* and *BFT* and increased flowering branch formation [74]. Thus, DNA methylation not only participates in the immediate response of plants to drought signals but may also contribute to long-term adaptation through stress memory and developmental regulation.

### 3.3. Salt Stress

During salt stress responses, DNA methylation not only is linked to changes in the expression of salt-responsive genes, but may also contribute to plant adaptation by affecting transposon activity and genome stability. In Arabidopsis, salt stress induces relatively limited methylation changes, with approximately 0.1% of cytosine sites showing differential methylation [75]. About one-third of these sites are located in transposable element (TE) regions, and although some salt-induced methylation changes can be maintained in subsequent generations, their inheritance may not be stable in all cases [76]. Plants with different genetic backgrounds exhibit distinct DNA methylation responses during salt adaptation. For example, cultivated beet (*B. vulgaris*) and its salt-tolerant wild ancestor sea beet (*B. vulgaris* subsp. *maritima*) display different CpG methylation patterns under salt treatment. Cultivated beet mainly shows reduced CpG methylation at salt-responsive gene loci, whereas wild sea beet exhibits predominantly hypermethylation, reflecting distinct epigenetic regulatory features associated with salt tolerance divergence [77]. In addition to genetic background, the expression patterns of DNA methylation regulators also show tissue specificity. In *Pyrus betulaefolia*, most DNA methyltransferase (PbeMTase) family members are highly expressed in roots and induced by salt stress, whereas their responses in stems and leaves are different [78]. In cotton, salt stress mainly coincides with increased expression of DNA methyltransferase C5-MTase genes in the leaves of diploid materials, whereas tetraploid upland cotton shows a predominant increase in expression in roots and stems [79].

Beyond these differences in methylation patterns, recent studies have further elucidated the specific mechanisms underlying DNA methylation-mediated responses to salt stress. In Citrus, polyploidization-enhanced salt tolerance is closely associated with methylation changes in the gene body region of *ACO1*. Tetraploid citrus and trifoliate orange promote *ACO1* expression and ethylene biosynthesis through CG demethylation in the *ACO1* gene body region and increased chromatin accessibility, which are linked to root development, maintaining K^+^/Na^+^ homeostasis, enhancing ROS scavenging, and improving salt tolerance in polyploids [80]. In the mangrove plant *Bruguiera gymnorhiza*, high-salt environments are accompanied by CHG and CHH non-CG hypermethylation in transposable element regions. The upregulation of methylation-related factors such as *CMT2*, *CMT3*, and *DRM2* may suppress abnormal transposon activation and maintains genome stability under high salinity conditions [81]. In addition, RdDM-mediated non-CG methylation remodeling represents an important regulatory mechanism in plant salt adaptation. In maize (*Zea mays*), *ZmKTF1* regulates CHH methylation levels in gene flanking regions through the RdDM pathway, suppressing excessive transcription of RBOH-related oxidative stress genes; loss of *ZmKTF1* reduces CHH methylation and weakens salt tolerance [82]. Under salt stress, rice enhances non-CG methylation of the MITE transposon near the *OsHKT1*;*5* promoter, accompanied by the presence of the methylation reader protein OsSUVH7 and its cooperation with OsBAG4-OsMYB106 in the activation of *OsHKT1*;*5* expression, Na^+^ retrieval, ion homeostasis, and salt tolerance [83]. Other epigenetic modifications also participate in salt stress responses and interact with DNA methylation regulation. In wheat, salt stress is accompanied by reduced global DNA methylation levels; demethylation of the *TaFLS1* promoter promotes its expression, while histone acetylation mediated by TaGCN5, including increased H3K9 and H3K14 acetylation, may jointly activate salt-responsive genes and enhances salt tolerance [84].

## 4. Perspectives and Conclusions

Previously reported DNA methylation responses vary among species and experimental conditions, including tissue type, developmental stage, stress intensity and duration, and methylation profiling methods (Table 1, Table 2 and Table 3), which limits direct comparisons of specific methylation responses across species. Nevertheless, these findings still provide an overall view of DNA methylation responses to different types of abiotic stress.

**Figure 2:**
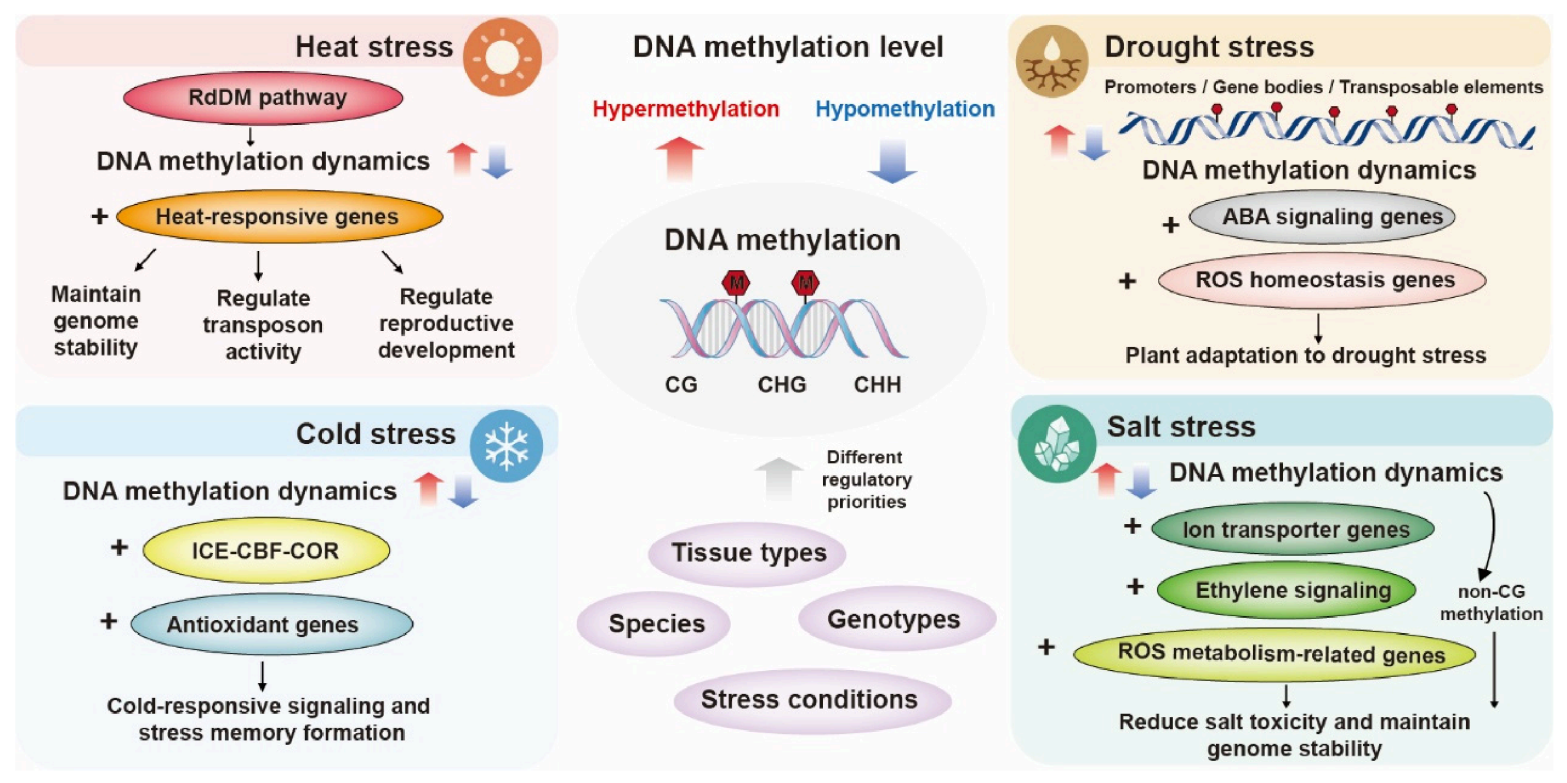
DNA methylation-mediated regulation of plant responses to abiotic stress. DNA methylation dynamics contribute to plant responses to heat, cold, drought, and salt stress, with distinct regulatory priorities under different stresses. Heat stress mainly involves RdDM-mediated methylation, genome stability, and reproductive development; cold stress is associated with ICE-CBF-COR signaling and stress memory; drought stress involves ABA signaling, ROS homeostasis, and root development; and salt stress is related to ion homeostasis, ROS metabolism, and genome stability.

In response to high-temperature, low-temperature, drought, and salt stresses, plants utilize DNA methylation-mediated epigenetic regulation to convert environmental signals into specific transcriptional responses; however, different stress types exhibit distinct regulatory priorities (Figure 2). Under high-temperature stress, DNA methylation may be primarily involved in maintaining genome stability and regulating reproductive development, through methylation remodeling at specific sites and genomic regions, particularly RdDM-mediated CHH methylation, which regulates transposon activity and the expression of heat-responsive genes [37,38,39,40,41,42,43,44]. Epigenetic variation occurs predominantly in reproductive organs, where methylation remodeling is closely associated with anther development, changes in fertility, and differences in heat tolerance [46]. In contrast, cold stress may involve the regulation of cold-responsive signaling and stress memory formation, with DNA methylation dynamics closely linked to the regulation of the ICE-CBF-COR pathway and antioxidant-related genes, and exhibiting marked differences among species and genotypes [50,51,52,53,54,55,56,57,58,59]. Some methylation marks could also persist beyond the stress period and contribute to stress memory [60,61]. Under drought stress, DNA methylation exhibits broad and dynamic regulatory patterns, with methylation remodeling distributed across promoters, gene bodies, and transposable elements and closely linked to drought-responsive processes such as ABA signaling, osmotic regulation, ROS homeostasis, metabolism, and root development, contributing to plant adaptation to drought stress through modulation of the methylation status of relevant genes [62,63,64,65,66,67,68,69,70,71,72,73,74]. In contrast, under salt stress, methylation regulation is more focused on ion homeostasis, ROS metabolism, and genome stability, reducing salt toxicity through the regulation of ion transporter genes, ethylene signaling, and ROS metabolism-related processes, while maintaining transposon silencing and genome stability through non-CG methylation [75,76,77,78,79,80,81,82,83,84].

It is noteworthy that, even under the same stress conditions, different species or genotypes may exhibit markedly different DNA methylation responses, or even opposite directions of methylation change. For example, beet and poplar show genome-wide demethylation after cold treatment, whereas apple shows no significant overall methylation change; cultivated beet shows CpG demethylation at salt-responsive gene loci under salt stress, whereas sea beet shows predominantly CpG hypermethylation [50,51,52,77]. These interspecific differences may be related to differences in the expression levels and stress responses of key DNA methylation regulators. Therefore, the stress responses of different species could not be assessed solely based on increases or decreases in DNA methylation, but should instead be evaluated by considering species, genotype, tissue, stress conditions, and specific genomic regions.

In addition to DNA methylation, RNA and histone modifications also participate in plant stress responses, indicating that epigenetic regulation in plants is not limited to a single type of modification. DNA methylation may act together with histone modifications in plant stress responses [85,86], whereas the RNA methylation modification m^6^A primarily affects post-transcriptional RNA stability, translation, and metabolism. For example, in *Arabidopsis thaliana* [49], apple [87], and rice [88,89], changes in m^6^A modifications and their associated regulatory factors have been linked to processes such as the CBF-COR cold-response pathway, ROS scavenging, and chloroplast function. Therefore, future studies could further examine plant stress adaptation from multiple epigenetic regulatory layers, including DNA methylation, histone modifications, and RNA methylation, to clarify the specific roles of different modifications under different stress types and physiological processes and thereby develop a more comprehensive understanding of the mechanisms underlying plant stress adaptation.

With the advancement of CRISPR technology, the CRISPR/dCas9 system has emerged as a powerful tool for epigenetic regulation. Since the dCas9 protein lacks DNA cleavage activity, it can be specifically targeted to genomic regions under the guidance of sgRNA and fused with DNA methyltransferases, demethylases, histone-modifying enzymes, or transcriptional regulators to achieve targeted remodeling of epigenetic states, providing new approaches for elucidating DNA methylation functions and generating novel epigenetic materials [90,91,92]. For example, in *Arabidopsis*, CRISPR/dCas9-mediated targeted methylation or demethylation of the *FWA* promoter, *FT* distal enhancer, and *SDC* promoter altered gene expression and resulted in changes in flowering time and leaf development phenotypes [93]. However, current applications of this technology are still limited by relatively low editing efficiency, insufficient stability of epigenetic modifications, and potential off-target effects, particularly in perennial crops and plants with complex genomes. In the future, DNA methylation-based precision epigenetic breeding is expected to enable the targeted improvement of key agronomic traits. By establishing stable and heritable epigenetic germplasm resources, this approach may facilitate the connection between environmental responses, gene expression regulation, and desirable trait formation, promoting the transition of epigenetic breeding from an auxiliary strategy in conventional breeding toward a precision strategy for germplasm innovation.

## Figures and Tables

**Table 2:** DNA methylation and epigenetic regulation in plants under drought stress.

Stress Type	Species	Tissue	Treatment	Method	Evidence Type	Methylation Change	Genes/Pathways Involved	References
Drought	*Morus alba*	Young leaf tissues	Soil moisture content 20–30%, 10 d	WGBS, RNA-seq, qRT-PCR	Expression correlation	Overall increase in genome-wide DNA methylation, mainly mCG increase	Autophagy, MAPK, AMPK, carbon metabolism, amino acid biosynthesis genes	[62]
Drought	*Linum usitatissimum*	Seedling leaf tissues	Absolute soil water content: 10% for 2 d, 70% rewatering, 10% after 5 d	MethylC-seq (WGBS), RNA-seq	Expression correlation	Dynamic changes in CG, CHG, and CHH methylation; more hypermethylated regions in drought-tolerant cultivar Z141	Carbohydrate/lipid metabolism, ABA signaling, osmotic regulation, antioxidant genes	[63]
Drought	*Populus tomentosa*	Functional mature leaf tissues	20–25% soil water content for 37 d, then rewatering to 80% for 33 d; 5-azaC treatment	WGBS, RNA-seq, 5-azaC treatment	Pharmacological perturbation	Overall decrease in DNA methylation levels; CHH most responsive	*PtoGATA9*, *LECRK-VIII.2*; transposon regions	[64]
Drought	*Oryza sativa*	Two-week-old hydroponic seedling	desiccation stress 3 h	WGBS, RNA-seq	Expression correlation	Extensive CHH demethylation in drought-tolerant Nagina 22; relatively limited methylation changes in drought-sensitive IR64	*bZIP*, *NAC*, *AP2*, *ZEP*, cysteine protease inhibitors	[65]
Drought	*Vigna radiata*	V1-stage seedling leaf tissues	Drought: withholding water until 39% relative soil water content	WGBS, RNA-seq, qRT-PCR	Expression correlation	Increase in CHH methylation; enrichment of hypermethylated DMRs in drought-tolerant line; decreased CHH methylation; hypomethylated DMRs in drought-sensitive	Carbohydrate metabolism, signal transduction, RNA metabolism genes	[66]
Drought	*Zea mays*	Root tissues	well-watered and water-stress (10% (*w*/*v*) PEG 8000 for 24 h)	MeDIP-seq, RNA-seq, sRNA-seq	Expression correlation	Sensitive genotype produces substantially more hypermethylated DMRs	Osmotic stress, oxidative stress, water/salt response, RNA silencing genes	[67]
Drought	*Triticum aestivum*	Seedling leaf tissues	Well-watered: 90% field capacity; mild stress: 50%; severe stress: 25%; 14 d	MSAP, qRT-PCR	Expression correlation	Genome-wide demethylation; promotes demethylation/activation of stress-responsive genes in drought-tolerant Bolani; genome-wide demethylation in drought-sensitive Sistan	*PEPC*, *BGlu*, *GST*, *LSD*, defense response genes mediated by ABA	[68]
Drought	*Zea mays*	Seedling leaf tissues	Relative leaf water content (RLWC): 98% control, 0% moderate drought, 58% severe drought	GWAS, McrBC-qPCR, Bisulfite sequencing, ChIP-qPCR, Arabidopsis mutant, RNA-seq, transgenic lines	Genetic/epigenetic validation	RdDM-induced CG and CHH hypermethylation	*ZmNAC111*; 82-bp MITE insertion	[69]
Drought	*Malus domestica*	Seedling leaves, root system, callus tissue	Hydroponic: 20% PEG8000 for 6 h; soil-grown: water withheld for 10–15 d; 5-aza treatment	Bisulfite sequencing, McrBC-qPCR, DAP-qPCR, apple transgenic lines	Pharmacological perturbation, genetic/epigenetic validation	CG, CHG, CHH hypermethylation via RdDM pathway	*MdRFNR1-1*; 430-bp MITE insertion	[70]
Drought	*Zea mays*	Seedling root tissues	100% soil water-holding capacity (control); 0% for 15 d (drought)	Bisulfite-PCR, RT-PCR	Expression correlation	CG/CHG demethylation and CHH hypermethylation in middle promoter region	*ZmEXPB2*	[71]
Drought	*Hordeum vulgare*	Seedling aerial parts	Hydroponic seedlings: well-watered control; water withheld for 10 d (drought)	Bisulfite-PCR, qRT-PCR	Expression correlation	Region-specific: CG/CHG increase, CHH decrease in distal regulatory region	*HvDME*	[72]
Drought	*Triticum aestivum*	Seedling leaf tissues	10% PEG preconditioning/15% PEG preconditioning/20% PEG preconditioning/direct treatment with 20% PEG for 72 h	Bisulfite-PCR, qRT-PCR	Expression correlation	CG/CHG demethylation in specific regions	*TaDME*, *TaMET2a/2b/3*, *TaP5CS*, *TaBADH*	[73]
Drought	Citrus (*Satsuma mandarin*)	Mature stem/shoot tissues	Mild drought, 13–15% for 5 months; 5-Aza treatment	WGBS, RNA-seq, 5-Aza treatment	Pharmacological perturbation	Genome-wide CHH hypermethylation	*CsDME*, *CsDML*, *FLC*, *BFT*	[74]

Note: Descriptive methylome refers to genome-wide or methylome profiling without direct functional validation; expression correlation refers to associations between methylation changes and gene expression without direct perturbation; pharmacological perturbation refers to studies using DNA methylation inhibitors such as 5-azacytidine or zebularine; and genetic/epigenetic validation refers to evidence obtained through genetic manipulation or targeted epigenetic editing.

**Table 3:** DNA methylation and epigenetic regulation in plants under salt stress.

Stress Type	Species	Tissue	Treatment	Method	Evidence Type	Methylation Change	Genes/Pathways Involved	References
Salt	*Arabidopsis thaliana*	Rosette leaves	50 mM NaCl every 4 d; 125 mM NaCl treatment	WGBS, WGS	Descriptive methylome	Relatively limited methylation changes; salt-induced methylation changes can be inherited	Transposable elements	[75,76]
Salt	*Beta. vulgaris*—cultivated beet vs. *B. vulgaris* subsp. *Maritima-* wild sea beet	Leaf tissues	Salt shock: leaves in 300 mM NaCl for 48 h; salt stress: gradually increased to 300 mM over 16 d, then maintained for 16 d	MSRE-qPCR, qPCR	Expression correlation	Cultivated mainly demethylation at salt-responsive loci; wild sea beet predominantly hypermethylation	Salt-responsive genes	[77]
Salt	*Pyrus betulaefolia*	Roots, stems, leaves	200 mM NaCl, 72 h	Bisulfite-PCR, qRT-PCR	Expression correlation	Most PbeMTase family members highly expressed in roots and induced by salt stress in root	PbeMTase family	[78]
Salt	*Gossypium raimondii*, *Gossypium arboreum*, *Gossypium hirsutum*	Roots, stems, leaves	Drought: water withheld for 6 d; salt: 200 mM NaCl for 2 d	qRT-PCR, VIGS	Expression correlation	Increased C5-MTase expression mainly in leaves of diploid materials, but predominantly in roots and stems of tetraploid upland cotton	C5-MTase genes	[79]
Salt	Citrus	Seedlings	200 mM NaCl, 15 d	WGBS, ATAC-seq, RNA-seq, Bisulfite-PCR	Expression correlation	CG demethylation in the ACO1 gene body and increased chromatin accessibility	ACO1; ethylene biosynthesis	[80]
Salt	*Bruguiera gymnorhiza*	Mature leaves	Hydroponic: low salinity 1.5%; high salinity 4%	WGBS, RNA-seq	Expression correlation	CHG and CHH hypermethylation in transposable element regions	*CMT2*, *CMT3*, *DRM2*	[81]
Salt	*Zea mays*	Aboveground tissues of seedlings	150 mM NaCl, 7 d	Map-based cloning, WGBS, RNA-seq, CRISPR-Cas9	Genetic validation	ZmKTF1 promotes CHH methylation in gene-flanking regions; loss of ZmKTF1 reduces CHH methylation	*ZmKTF1*; RBOH-related oxidative stress genes	[82]
Salt	*Oryza sativa*	Seedling roots, leaves	100 mM NaCl, 5 d	Bisulfite-PCR, CRISPR-Cas9, transgenic complementation	Genetic validation	Increased non-CG methylation of MITE near the OsHKT1;5 promoter	*OsHKT1*;*5*; *OsSUVH7*; *OsBAG4-OsMYB106*; MITE transposon	[83]
Salt	*Triticum aestivum*	Seedling roots, leaves	200 mM NaCl	MSAP, WGBS, Bisulfite-PCR, ChIP-qPCR	Expression correlation	Salt-induced global DNA hypomethylation, TaFLS1 promoter demethylation	TaFLS1, TaGCN5, salt-responsive genes	[84]

Note: Descriptive methylome refers to genome-wide or methylome profiling without direct functional validation; expression correlation refers to associations between methylation changes and gene expression without direct perturbation; pharmacological perturbation refers to studies using DNA methylation inhibitors such as 5-azacytidine or zebularine; and genetic/epigenetic validation refers to evidence obtained through genetic manipulation or targeted epigenetic editing.

## Data Availability

No new data were created or analyzed in this study. Data sharing is not applicable to this article.
